# Modelling health and economic impact of nutrition interventions: a systematic review

**DOI:** 10.1038/s41430-022-01199-y

**Published:** 2022-10-04

**Authors:** Mariska Dötsch-Klerk, Maaike J. Bruins, Patrick Detzel, Janne Martikainen, Reyhan Nergiz-Unal, Annet J. C. Roodenburg, Ayla Gulden Pekcan

**Affiliations:** 1 Unilever Foods Innovation Centre, Wageningen, The Netherlands; 2 DSM Nutritional Products, Kaiseraugst, Switzerland; 3 Novartis, Basel, Switzerland; 4 University of Eastern Finland, Kuopio, Finland; 5 University of Hacettepe, Ankara, Turkey; 6 HAS University of Applied Sciences, s-Hertogenbosch, The Netherlands; 7 University of Hasan Kalyoncu, Gaziantep, Turkey; 8 Present Address: Unilever Foods Innovation Centre, Wageningen, Bronland 14, 6708 WH, The Netherlands

**Keywords:** Population screening, Computational models

## Abstract

Diet related non-communicable diseases (NCDs), as well as micronutrient deficiencies, are of widespread and growing importance to public health. Authorities are developing programs to improve nutrient intakes via foods. To estimate the potential health and economic impact of these programs there is a wide variety of models. The aim of this review is to evaluate existing models to estimate the health and/or economic impact of nutrition interventions with a focus on reducing salt and sugar intake and increasing vitamin D, iron, and folate/folic acid intake. The protocol of this systematic review has been registered with the International Prospective Register of Systematic Reviews (PROSPERO: CRD42016050873). The final search was conducted on PubMed and Scopus electronic databases and search strings were developed for salt/sodium, sugar, vitamin D, iron, and folic acid intake. Predefined criteria related to scientific quality, applicability, and funding/interest were used to evaluate the publications. In total 122 publications were included for a critical appraisal: 45 for salt/sodium, 61 for sugar, 4 for vitamin D, 9 for folic acid, and 3 for iron. The complexity of modelling the health and economic impact of nutrition interventions is dependent on the purpose and data availability. Although most of the models have the potential to provide projections of future impact, the methodological challenges are considerable. There is a substantial need for more guidance and standardization for future modelling, to compare results of different studies and draw conclusions about the health and economic impact of nutrition interventions.

## Introduction

Malnutrition involving micronutrient deficiencies, undernutrition, overweight and obesity, as well as non-communicable diseases (NCDs) are universal public health and economic problems [1]. NCDs, such as cardiovascular diseases (CVD), cancer, respiratory diseases and diabetes are the leading causes of mortality and morbidity globally and as such are the main contributors to health costs worldwide [2]. Many of these diseases result from unhealthy diets and might be preventable [1]. The global community continues dealing with multiple burdens of malnutrition despite the various steps relevant stakeholders worldwide have been taking over the past few decades towards improved nutrition and reduction in associated health burdens [3, 4]. Global diet and nutrition surveys show that many populations still consume high amounts of discretionary foods high in sugars, saturated fat, salt and not enough fruits, vegetables, legumes and whole grains [5–10]. The health impact and economic burden of insufficient or unhealthy diets is substantial [4]. Other populations face a serious burden of malnutrition i.e. childhood stunting, anaemia of women in reproductive age, micronutrient deficiencies and/or obesity in all ages [3, 4]. Studies have shown that micronutrient deficiencies such as vitamin D [11], folate [12] and iron [13] are globally highly prevalent [14, 15] and pay a heavy toll on economies.

Nutrition interventions and food-based global strategies aim to change nutrient intakes either by changing composition of foods or by changing consumer behaviour and by providing support for making healthier food choices. Many countries have reduced population-wide salt intake in recent years through regulations on salt content in processed foods, labelling of processed and prepared foods, public education and by increased engagement of the food industry [16]. These salt reduction interventions are regularly evaluated using salt modelling studies, and population-wide salt reduction programs are considered a cost-effective strategy [17]. Regular consumption of sugar-based beverages is associated with excess weight gain and a number of co-morbid conditions, including diabetes, CVD and dental caries [18]. Taxation of sugar-based beverages is often suggested as a regulatory approach to address population weight gain, and also in this case modelling studies are being performed to evaluate the potential health and economic impact. Large-scale food fortification is an emerging area of interest for improving nutrient intakes, especially in more vulnerable populations [10]. While micronutrient public health programs focus on a broad set of micronutrients including also vitamin A, D, folic acid, zinc, iron, and iodine [3], the modelling studies in this area are mostly focusing on vitamin D, folic acid, and iron.

To support policy-making, modelling approaches are frequently used to estimate the potential long-term impact of nutrition interventions on health and economic outcomes [19]. Health and economic modelling in the domain of public health nutrition is still in its infancy. There is neither a recommended best practice model nor a clear guidance for rating these models. The objective of this review was to obtain insights into the different modelling approaches that are currently used to estimate the health and/or economic impact of nutrition interventions. As an example, we selected modelling studies aiming at reducing population intake of salt and sugars or increasing intake of micronutrients. Therefore, in this paper, we systematically reviewed and evaluated modelling studies using expert pre-defined quality and applicability criteria. These insights may serve recommendation for best practice models in nutrition economics.

## Methods

### Literature search strategy

The protocol of this systematic review has been registered with the International Prospective Register of Systematic Reviews (PROSPERO: CRD42016050873). The final search was conducted on PubMed and Scopus electronic databases in August 2020. Search strings were developed for salt/sodium (RNU), sugars (MDK), vitamin D, folic acid and iron (MB) (Supplemental information 1). Per subitem two reviewers independently checked the abstracts of all papers initially identified using the eligibility criteria mentioned in the next paragraph (Salt: JM, AGP; sugars: MD, PD; vitamin D, folic acid and iron: AJCR, MJB). If the different reviewers did not agree, the original publication was consulted, and a consensus was reached via discussions. After completion of the abstract screening authors screened the full text of the articles for inclusion and appraisal (Salt: JM, AGP, RNU; sugars: MD, PD; vitamin D, folic acid and iron: AJCR, MJB).

### Eligibility criteria

Studies that modelled the impact of modifying nutrient intake on public health and/or economic outcomes due to intervention (e.g., estimating potential changes in risk factor, disease incidence, disease burden, quality-/disability-adjusted life years, or health care costs) were selected as eligible modelling studies. The search was limited to articles published in English from 2006 until August 2020. Furthermore, additional filtering criteria were applied to restrict the number of irrelevant studies (Supplemental information 1).

**Fig. 1:**
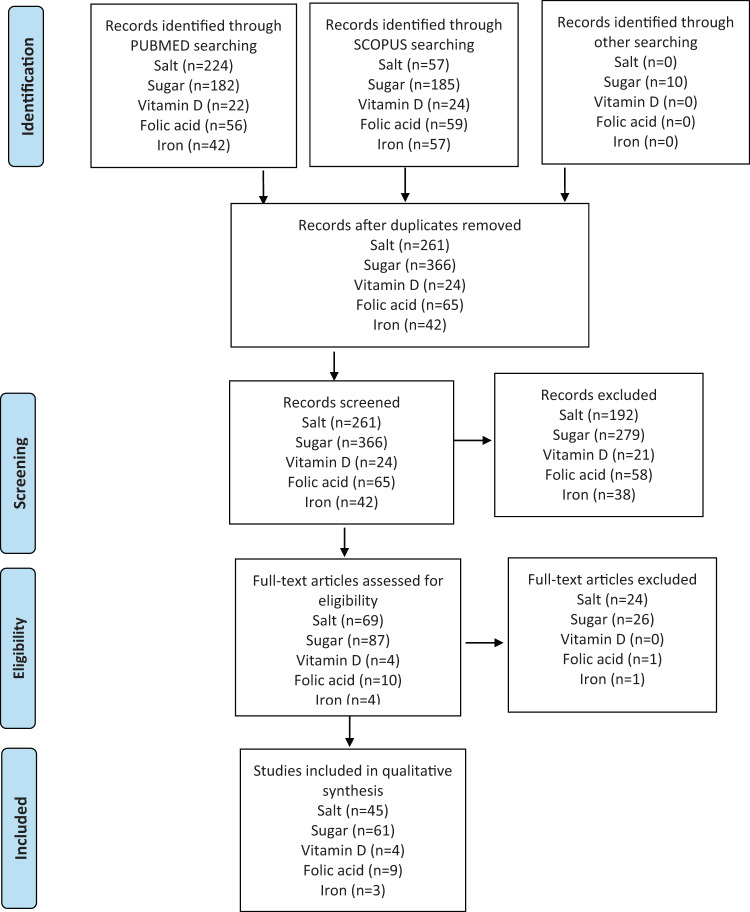
The flow chart of the study selection.

The Prisma flowchart was used to create an overview of the screening process and included studies (Fig. 1).

### Study description and evaluation criteria

Models were classified according to type of applied modelling approach, such as decision tree, comparative risk assessment, Markov models, and discrete event simulation. More information about these different types of models is provided in Table 1 [20]. In addition, the different modelling scenarios and outcome focus of the studies were described.

**Table 1:** Model classification and explanation*.

Type	Explanation
Decision tree	A flowchart-like structure, where the probability of events and their consequences are presented in a graphical form.
Comparative risk assessment	An analytic process of evaluating and ranking the different factors that contribute to a particular outcome
Markov model	Transition model with discrete health states w/o probabilistic sensitivity analysis (PSA)
Markov chain model	Transition model with constant transition probabilities (vs. Markov process model, where transition probabilities are enabled to change over time)
Markov individual model	“Markov microsimulation model”, where a number of single patient run through the model instead of patient cohorts, which is usually a case in Markov models
Discrete event modelling	Model outcomes as a discrete sequence of events in time.
Agent-based approaches	Enable to simulate the actions and their consequences while taking into account the interactions of autonomous agents (both individual and different groups) with a view to assessing their effects on the system as a whole

*Adapted from Briggs et al. 2016 (20).

To be able to evaluate the models found in different publications, a set of draft criteria was developed, covering both scientific- and applicability aspects. These were based on the questionnaire to assess relevance and credibility of modelling studies for informing health care decision making as defined by the International Society for Pharmacoeconomics and Outcomes Research (ISPOR) [21]. Other publications were used for guidance [22–24]. The criteria were not meant to be interpreted as a scorecard or checklist but served to create a good overview and help the discussion on what appropriate models would be. These criteria were extensively discussed with different stakeholders in the field (public health scientists, health economics, policy makers, and industry members) and modeling experts during an International Life Sciences Institute (ILSI) Europe workshop on ‘Identifying Preferred Approaches for Quantifying the Health and Economic Impact of Modifying Nutrient Intakes’ (April 6–7, 2017, Brussels, Belgium).

After the workshop, the criteria were adapted where necessary to reflect the experts’ views and the adapted criteria were used to evaluate all individual studies. The final criteria used to evaluate individual modelling studies are shown in Table 2. The scientific aspects evaluated by the authors included the quality of the input data, robustness, and transparency of the model, as well as inclusion of sensitivity/uncertainty analyses. The applicability aspects evaluated by the authors included data needs, accessibility of the model, research funding and if there could be a conflict of interest (Table 2).

**Table 2:** Criteria used to evaluate individual modelling studies.

Criteria		Options/rating
Scientific	Quality of data input Are the data used to estimate impact (e.g. associations between exposure and risk factors, or risk factor and disease, cost input) based on relevant evidence?	Low: Model inputs are derived from multiple sources with varying quality. Many assumptions based on expert opinions. Medium: Model inputs are derived from multiple sources with good quality. Only some assumptions are based on expert opinions. High: Majority of model inputs are derived from small number of studies or a single study with “high-quality” data providing information. e.g. about the associations between exposure factors, outcomes, quality of life, and health care utilization.
	Model robustness Is the model structure consistent with both a coherent theory of health condition being modeled and with available evidence regarding causal linkages between variables?	High robustness: if using one of the recognized models (e.g. RIVM, PRIME or IMPACT). Medium robustness: if it concerns a model based on a consistent theory of health condition (e.g. clear health consequences hypertension leading to CVD) and when estimated results are “validated” or “compared” with other estimates. Low robustness: if none of the above
	Transparency Are all details/assumptions of the modelling revealed or is the model a black box?	High transparency: if model equations, table with input parameters (and underlying distributions) and some “flow chart” is provided Medium transparency: if only a flow chart is provided Low transparency: if none of the above.
	Sensitivity/uncertainty analysis Are assumptions tested using sensitivity analyses?	Deterministic: Reports deterministic sensitivity analyses, either one-way sensitivity analysis (varying one factor at the time) or multiway (varying several factors simultaneously). Probabilistic: Reports probabilistic sensitivity analysis based on (second-order) Monte Carlo simulation to evaluate total parameter uncertainty (i.e. imprecision of parameters). Microsimulation (patient-level) is applied to evaluate the role of “random variation” on final outcomes. Different models: All previous + model structure uncertainty (i.e. different model structures are tested). Different survival models are tested to study sensitivity of applied model on final outcomes. None/Not applicable: If none of the above.
Applicability	Data needs What are the data needs in order to be able to run the model?	Little: The model runs on relatively little and readily available epidemiological data. Modest: The model needs less readily available data on incidence, prevalence, and mortality by risk-factor status (e.g., for smokers and nonsmokers) or on transitions between risk-factor states. Considerable: The model needs data on longitudinal panel/cohort data in order to be able to perform microsimulation.
	Accessibility Are models accessible for use by others?	Open source model: Established open source model, accessible for use by others. Reproducible: All model parameters and software used are available for use by others to reproduce the model. Not accessible: Established or self-built model, not accessible by others.
Other	Source of funding How has the study been funded?	Public: Funded by public money only. Private: Funded by private sector. Both public and private: Funded by public and private money. Not clear: Funding source not mentioned. No funding: Not founded by public or private money.
	Potential conflict of interest Is the study subject to potential conflict of interest?	No: Study performed by a research institution/academia. Authors clearly state the funder(s) had no role in study design, data collection and analysis, decision to publish, or preparation of the manuscript. Yes: Involvement and funding by a party that has a clear personal or financial interest in the outcome of the results. Not clear: Authors do not clearly state that the funding body had no role in the study.

## Results

### Study selection

Figure 1 illustrates the PRISMA flowcharts for the search and review process. In total, 174 articles were screened based on full text. Totally 122 publications were included for critical appraisal: 45 for salt/sodium, 61 for sugars, 4 for vitamin D, 9 for folic acid, and 3 for iron. In total, 52 articles were excluded after both abstract and full-text screening, as they were considered not eligible because of in- and exclusion criteria applied criteria: 24 for salt/sodium, 26 for sugars, 1 for folic acid and 1 for iron (Fig. 1). An overview of all studies and models included in the review is provided in Table 3 and evaluation results in Table 4.

**Table 3:** Overview of the type of models used in publications.

Model type	Salt (*n* = 45)		Sugar (*n* = 61)		Vitamin D (*n* = 4)		Folic acid (*n* = 9)		Iron (*n* = 3)	
	*n*	References	*n*	References	*n*	References	*n*	References	*n*	References
Decision tree	2	Palar & Sturm, 2009*;* Dall et al. 2009	1	Hendriksen, et al., 2011			7	Llanos et al. 2007; Hoekstra et al. 2008; Jentink et al. 2008; Rabovskaja et al. 2013; Kancherla 2018; Hoddinott 2018; Saing et al. 2019	1	Plessow 2016 (refers to Plessow 2015; Wieser 2013)
Comparative risk assessment	22	Qin et al. 2009; Cobiac et al. 2010; Rubinstein et al., 2010; Barton et al. 2011; O’Flaherty et al. 2012; Dodhia et al. 2012; Kontis et al. 2014; Kontis et al. 2015; Bruins et al. 2015; Cobiac&Tam et al. 2017; Caro et al. 2017; Fitzgerald et al. 2018; Moreira et al. 2018; Hendriksen&Over et al. 2018; Aminde et al. 2019; Labonte et al. 2019; Briggs et al. 2019; Goiana-da-Silva et al. 2019; Saha et al. 2019; Blakely et al. 2020; Marklund et al. 2020; Nilson et al. 2020	19	Fletcher et al., 2010; Finkelstein et al., 2010& 2013; Dharmasena et al. 2012; Manyema et al., 2014; Härkänen 2014; Lin 2011; Ruff et al. 2015; Gustaven et al. 2013; Meijer et al., 2015; Briggs et al. 2017; Cobiac et al. 2017; Ginsberg et al., 2017; Penalvo et al. 2017; Schwendicke et al. 2017; Vecino-Ortiz et al. 2018; O’Neill et al. 2019; Scheelbeek et al. 2019; Zheng et al. 2019; Goiana da Silva et al. 2020.	1	Sandmann et al., 2015				
Markov (deterministic)	7	Basu et al. 2012; Coxson et al. 2013*;* Nghiem et al. 2015; Islek et al. 2016; Nghiem et al. 2016; Cobiac&Scarborough 2017; Hendriksen&Geleijnse et al. 2017	27	Haby et al., 2006; Manyema et al., 2015& 2016; Mekonnen et al., 2013; Wang et al. 2012; Long et al. 2015; Ma et al. 2016; Sanchez-Romero et al. 2016; Veerman et al. 2016;; Barrientos-Gutierrez, 2017; Basto-Abreu, 2019; Bourke et al. 2018; Amies-Cull et al. 2019; Cleghorn et al. 2019; Crino et al. 2017; Jevdjevic et al. 2019; Blakely et al. 2020; Huse et al. 2019; Kao et al. 2020; Lal et al. 2017; Lal, 2020; Lee, 2020; Nomaguchi et al. 2020; Salgado et al. 2020; Sowa et al. 2018; Wilde, 2019						
Markov chain	5	Martikainen et al. 2011; Scarborough, Allender et al. 2012; Scarborough, Nnoaham et al. 2012; Hendriksen et al. 2015; Erkoyun et al. 2016	3	Briggs et al, 2013a; Briggs et al. 2013b; Cobiac et al. 2016						
Markov individual (microsimulation)	1	Pearson-Stuttard et al. 2018	6	Basu et al., 2014; Basu et al. 2020; Gortmaker et al. 2015; Grummon et al. 2019; Lee et al. 2020; Wilde et al. 2019	3	Ethgen et al., 2016;Hiligsman et al. 2015; Hiligsman et al. 2018			1	Sharieff 2008
Discrete event simulation (DES)	8	O’Keefe et al., 2013; Mason et al. 2014; Collins et al. 2014; Ni Mhurchu et al. 2015; Moreira et al. 2015; Hughes et al. 2015; Gillespie et al., 2015; Kypridemos et al. 2017	3	Pearson-Stuttard et al. 2017; Huang et al. 2019; Long et al. 2019						
Agent-based approaches			1	Lee et al. 2018						
Not clear			1	Urwannachotima et al. 2020			2	Bentley et al., 2009; Dalziel et al., 2010	1	Fiedler 2013

**Table 4:** Evaluation of studies according to the determined final criteria.

Criteria items	Salt (*n* = 45)	Sugar (*n* = 61)	Vitamin D (*n* = 4)	Folic acid (*n* = 9)	Iron (*n* = 3)
	*n*	*n*	*n*	*n*	*n*
Scientific					
Quality of data input					
High quality	38	22	0	1	0
Medium quality	5	33	4	6	0
Low quality	2	6	0	2	3
Model robustness					
High	29	18	0	0	0
Medium	16	36	4	3	1
Low	0	7	0	6	2
Transparent					
High	37	36	0	4	0
Medium	6	17	1	2	0
Low	2	8	3	3	3
Sensitivity/uncertainty analysis					
Different models	20	0	0	0	0
Probabilistic	22	43	0	2	1
Deterministic	3	15	4	6	2
None/Not applicable	0	3	0	1	0
Applicability					
Data needs					
Considerable	24	21	3	0	0
Modest	11	34	0	0	0
Little	10	6	0	6	3
Accessability					
Open source model	0	0	0	0	0
Reproducible	33	34	0	1	1
Not accessible	12	27	4	8	2
Source of funding					
Public funding	36	50	1	9	0
Private sector	2	1	0	0	2
Public and private	4	1	2	0	1
Not clear	3	5	1	0	0
Not funding	0	4	0	0	0
Conflict of interest					
No	34	54	21	9	1
Yes	7	2	1	0	2
Not clear	4	5	1	0	0

### I. Studies modelling health and economic impact of salt reduction interventions

An overview of the studies included and models used for modelling the impact of salt reduction is shown in Table 3 and evaluation results in Table 4 [9, 25–68]. More detailed information about the individual studies included can be found in the supplemental information.

### Intervention scenarios modelled

The studies included in the review focused on interventions to reduce salt/sodium intake to improve public health [25, 28, 29, 35, 36, 39, 40, 44, 50, 56, 57, 59–61, 64, 65]. The different types of scenarios modelled included sodium/salt reformulation [9, 26, 27, 31, 32, 34, 42, 44, 48, 66, 68], taxation [27, 32, 38, 43, 53], labelling (front-of-pack) of food packaging [44, 66], health promotion/awareness campaigns or dietary counselling [26, 27, 37, 46, 48, 68].

### Applied modelling approaches

As presented in Table 3, Markov-type (*n* = 13), comparative risk assessment (*n* = 22) or discrete event simulation (*n* = 8) models were most frequently used to model the health and economic consequences of different salt reduction interventions. In some cases (*n* = 2), also a simple decision tree modelling approach was applied. Some papers were published by the same research groups, but there was no research group dominating the publications on salt modelling using one and the exactly same model.

### Outcome focus

Health outcomes in the assessed studies were mainly measured by the number of CVD cases or deaths averted. In some cases, models were also applied to estimate the incidence and prevalence of CVD events and mortality, impact on quality of life and associated with their cost-effectiveness or cost-saving outcomes [9, 25–27, 29, 32, 33, 35, 38, 39, 42, 43, 45, 48, 49, 51–55, 57, 65, 66]. The rest of the assessed salt reduction models assessed the simultaneous impact on both the length and the quality of life i.e. QALYs (Quality-Adjusted Life Years) gained or DALYs (Disability-Adjusted Life Years) avoided or prevented, and health care costs saved [32, 39, 48, 49, 54, 55, 65, 66]. Literature indicates that interventions mainly focused on a reduction of salt intake in the whole population, like salt reduction in processed foods, mass media campaigns, and taxes related outcomes are evaluated for cost-effectiveness in the prevention of hypertension [31, 36, 40, 42, 51, 56, 57].

### Quality of input data

The salt related modelling studies, simulating the natural course of the diseases (e.g., from healthy to CVD, cancer, etc.) and dietary intake of salt consisting foods, need sufficient available input data [64]. In general, input data were obtained from chronic disease incidence and risk factors surveys to estimate the effect of various salt reduction policies on mortality, and on the length and the quality of life [26, 33, 35, 46, 57, 64]. In this present review, the majority (*n* = 38) of the selected studies were considered to have high quality input data that are representative for the population studied, while other studies were considered to have medium (*n* = 5) and low-quality (*n* = 2) input data (Table 4). Insufficient quality of the data typically occurred due to lower quality of input data sources, such as dietary intake surveys and integrated national health system interventions [27, 36, 39, 60, 64]. In addition, the lack of country-specific cost and quality of life input data, or use of simplified assumptions to reduce the complexity of the study were considered to impact on the quality of input data [27, 52].

### Robustness

Most salt modelling studies were scored as highly robust (Table 4). In particular, the robustness of the models was based on the reported reductions in salt intake. However, in some of these studies, salt levels in foods, changes in consumer knowledge, or individual attitudes and behaviours possibly impacting on consumption patterns were not estimated, which might affect the interpreted strength of the data [52, 64]. Rubinstein et al. [54] reported that effects of interventions can have different effects in subpopulations with different socio-economic statuses, but most studies did not consider these socio-economic differences.

### Transparency

Most reviewed studies were considered to have a high transparency (Table 4) [28, 29, 31–34, 36, 39, 41–46, 50, 51, 54–56, 60, 65, 67].

### Sensitivity analyses

In the studies on salt, in general long-term time horizons were applied to ensure that all expected relevant changes in cost and health outcomes are considered in analyses. This, naturally, increases uncertainty associated with the modelled health and economic outcomes. To address this temporal and the other sources of uncertainty different approaches are needed. In the present review, one-way sensitivity and scenario analyses were the most reported approaches to assess the robustness of the results. In addition, probabilistic sensitivity analysis, which could be considered as a state-of-art method, was also a commonly applied approach (Table 4) [28, 29, 31–34, 36, 39, 41–46, 50, 51, 54–56, 60, 65, 67].

### Data needs

The modelling studies showed substantial differences in data needs (Table 4). In many cases, the input data used in the modellings for salt intake reduction were derived from national health and dietary intake surveys [26, 27, 36, 39, 51, 52, 56–60, 64, 67], national vital statistics on mortality data for CVD, stroke and diet-related cancers and myocardial infraction, hypertension or stroke risks [26, 36, 39, 52, 56, 57, 59–61, 67, 68] and cost data from annual food cost surveys and reference costs collection guidance, previously published studies, and economic evaluation [26, 36, 39, 51, 68]. Generally, Markov-type and discrete event simulation models required more input data comparing to other modellings [25, 69].

### Accessibility

Most of the studies’ models are accessible (Table 4). However, models are usually complicated to use as special knowledge and training is needed [9, 25–27, 36, 39, 40, 52–54, 58–63, 67].

### Source of funding

In three of the published papers, the source of funding was not clear (Table 4). Among those that declared funding source, around half of the modelling studies were publicly funded, while a few were funded by either private sector or by both public and private sector. Generally, it was declared that the funders had no role in study design, data collection and analysis, decision to publish, or preparation of the manuscript.

### Conflict of Interest

A minority of the studies showed an interest conflict. Among these studies, the most common conflicts of interests were that authors have been employed by companies and/or the work was funded by companies, work was supported by a research grant from other stakeholders, consultation services, or being a shareholder. However, in 4 of the studies the conflict of interest was not clearly stated, but the author contributions were given, and no clear indication was determined that results were affected (Table 4).

### II. Studies modelling health and economic impact of sugar reduction interventions

An overview of the 61 studies included and models used for modelling the impact of sugar reduction is shown in Table 3 and the evaluation results in Table 4 [39, 42, 43, 70–129]. Details for the individual studies are provided in the supplementary information.

### Intervention scenarios modelled

Five studies estimated the impact of a reduction in sugar-based beverages or sugar intake [80–82, 103, 119]. One study modelled the impact of mandatory sugar reformulation [71]. One study [83] evaluated the impact of replacing beverages sweetened with sugar by beverages sweetened with artificial sweeteners on body mass index (BMI).Three studies evaluated the impact of sugar labeling [56] or warning signs [72, 124]. One study evaluated the impact of an increase in price of snacks [74]. Some studies evaluated the impact of removing [70] or discouraging television advertising or price promotion [129] of high sugar foods and beverages. [71] Two studies evaluated the impact of a ban of sugar-based beverages from the workplace [75] or from a national supplemental assistance program [76]. A few studies evaluated the impact of more comprehensive programmes that included reformulation, portion size reduction, price increase or changes in market share [77–79]. However, the large majority evaluated the potential impact of sugar tax on sugar-based beverages. The studies covered different global regions, and most were published only in the last 2–3 years. Most of these scenarios were more complex than simply evaluating the impact of product reformulation as it included a behavioural component and assumptions on potential replacements for the taxed or discouraged foods and beverages.

### Outcome focus

Approximately half of the studies investigated the impact of the modelling scenarios exclusively on health outcomes. The other half also evaluated the economic impact. There is large variation in the way the impact of sugar reduction on health and/or economic impact is modelled. The majority of the studies just modelled the impact of sugar intake reduction via weight change on obesity and/or diabetes risk and therefore may have underestimated the overall health impact [71, 72, 74, 77, 82, 84–98, 124, 129]. Some studies, in particular the ones published the past few years, looked at impact on multiple diseases, DALYs, QALYs or HALYs (Health-Adjusted Life Years) [42, 70, 75, 76, 79, 104–107, 109, 128]. Only a few studies included intermediary risk factors in the model such as blood pressure, cholesterol [39, 103, 108, 111], or glycemic load [112, 113] in addition to weight/BMI or included diabetes as a risk factor for CVD [39, 73, 81, 82, 111, 128] During the last 3 years, also several studies looking at the impact of a reduction in sugar intake on dental caries and/or oral care have been published [77, 78, 114, 115].

Economic outcomes mainly included health care cost savings or cost-effectiveness. Some studies also looked at tax revenues. [76, 84, 102, 109, 110, 125, 126] Two studies focused on income disparities [74, 101] One study looked also at changes in consumer spending [126] and one study at impact on productivity [92]. In most studies, health care costs included direct costs of hospital days and outpatient consultations, but studies did not consider incremental savings for preventing CVD events or other conditions leading to a potential underestimation of savings [99, 116]. However, studies may not have included all costs and revenues of the intervention which may have lead to under or overestimation of the economic impact [99, 111].

### Applied modelling approaches

An overview of the models used for modelling the impact of sugar intake reduction is shown in Table 3. Many of studies have used risk assessment models. However, even more studies have used established Markov models (*n* = 35), like PRIME [79, 96, 97, 103], the CVD Policy model [76, 108, 129], the US Impact Food Policy Model [101, 128], CVD PREDICT [106], ACE model [71, 90, 100, 105, 107, 119, 129] or Choices model [76, 104, 109]. The more complex models like agent based or discrete event modelling approaches were less often used. Some papers were published by the same research groups, but there was no research group dominating the publications on sugar modelling using one and exactly the same model.

### Quality of data input

Most studies were considered to have good quality input data that are representative for the population studied (Table 4). However, there were also studies where the quality of data input was rated as medium or even low. The accuracy of the intake data is key for the quality result. Most studies have used 24h-recall data, which are most reliable. However, various studies relied on self-reported intake data [75, 99, 108, 116, 127] or food frequency data [82, 97] which probably has led to underestimation of the true intake at baseline. Other studies have used purchase data (at household level) as a proxy for individual intake [74, 84, 85, 91, 98, 112, 117, 118, 124], but which may not reflect true intakes due to intrahousehold heterogeneity in terms of consumption.

Most studies have used good quality epidemiological data to model the impact of sugar intake on risk factors and/or health outcomes. Nevertheless, not all studies used the same data for estimating the impact on calorie reduction on weight or diabetes risk. For instance, some of the studies have used the formula of Christiansen and Garby [79, 83, 94, 96, 97] for the association between energy intake and BMI, whereas others have used the estimates of Swindburn [90, 99, 116, 127] or the equations of Hall [42, 72, 89, 95, 107, 112, 113, 119].

As shown earlier, most studies have modeled the impact of interventions involving a behavioral component rather than sugar reduction through reformulation. This implies more complexity as it requires an additional step or assumptions in the model, i.e., quantifying the impact of a behavioral intervention on intakes either by data or assumptions. There is a lack of implementation research about the real impact of behavioral interventions like taxes or removing TV ads on intakes. Therefore the true impact of these interventions is uncertain [70, 110].

Studies evaluating the impact of sugar tax also need to include estimates on price elasticities to account for substitution by other beverages and foods. Although there are some data on price elasticities of sugar-based beverages published, these types of data are not available for every population studied, and assumptions had to be made in various studies. In general, two approaches to estimate these behavioral changes were used. On one side there were the “econometric approaches” based on very large consumer panel data, using complex functions to estimate directly the different elasticities [42, 43, 74, 77, 84, 85, 88, 95, 98, 104, 107]. The other group of studies used group specific and general price elasticity functions derived from literature [72, 73, 76, 89, 90, 92–94, 96, 97, 105, 106, 114, 116, 120–122, 125, 127]. In addition, price elasticity could vary per socio-economic status and geographic location [96, 97], which was taken into account to at a certain extend by several studies [43, 73, 74, 89, 94–97, 101, 105–107, 113, 121]. It is also not clear, if tax was always passed on to the consumer for 100%, but in their baseline scenario most studies assumed a 100% pass on rate. Several studies, however, did vary with pass on rates in sensitivity and/or uncertainty analyses [39, 73, 77, 94–96, 99, 100, 103, 105–107, 114, 120, 127].

A number of studies, mainly the studies published the past few years [43, 72, 74, 77, 82, 84, 88, 90, 94, 105, 107, 123, 126, 128], did account for substitution of sugar-based beverages with other beverages. However, switches to other foods could also occur and so affect results. Overall, most studies did not account for any form of substitution, which could have led to an overestimation of the impact of the tax, price increase or label.

### Robustness

As mentioned, a large part of the studies has used risk assessment models, whereas most studies have used established Markov models like the CVD Policy Model, PRIME, IMPACT, ACE or Choices (Table 4). Most articles provided a clear and elaborate description of the model and were based on a coherent theory of the health condition being modelled. In some publications [70, 78, 80, 82, 86, 91, 126], the model structure was not very clear, and therefore robustness rated as low.

### Transparency

Most studies provided a clear and elaborate description of the structure, model parameters and assumptions, either in the article itself or in supplemental files, or referred to another publication where details can be found (Table 4). In a few studies, the model structure was clear from a flowchart but details on model parameters and assumptions was lacking. In eight studies the information was too limited to be able to properly evaluate the model [70, 80, 82, 94, 114, 115, 123, 126].

### Sensitivity analyses

All studies did some form of sensitivity or uncertainty analyses (Table 4), except for 3 studies [78, 83, 91]. Several studies performed only deterministic sensitivity analyses, evaluating different tax percentages or applied variations in other model parameters like price elasticity, pass-on rates, BMI trends or discount rates. Most of the studies performed probabilistic analyses using Monte Carlo simulation, using different numbers and type of parameters.

### Data needs

Most of the models showed at least modest or even considerable data needs (Table 4). Results clearly show that the more complex the model, the more data is needed. In general, the comparative risk assessment models required less data than Markov, discrete events or agent-based models.

### Accessibility

For many studies, models were not accessible, and publications did not provide enough information to reproduce the model (Table 4). However, most of the models for which sufficient details were published, and which were also rated as highly transparent, could be reproduced. None of the studies used an open-source model.

### Source of funding

Most of the models were funded by public money (Table 4). Funders mainly included national health associations. A few studies were partly funded by governmental bodies [80, 109]. This could probably be explained by the fact that sugars studies mainly evaluated the impact of policy interventions rather than food reformulation. In some publications the funding source was not provided [70, 83, 93, 113, 120] or was stated that funding was not applicable [72, 77, 78, 122].

### Conflict of interest

In most studies there was no sign of potential conflict of interest (Table 4). Several publications clearly stated that the funder of the study had no role in the design, execution and reporting of the study. [39, 42, 72, 79, 81, 89, 91, 99, 101, 103–105, 107, 111–113, 115, 116, 123, 128] In some of the studies, authors declared to have links with the private sector [73, 101, 106, 112, 120], or potential conflict of interest was not specified [70, 72, 75, 95, 97], but there was no indication of conflict of interest related to the study. In two studies [100, 126] there seemed to be a potential conflict of interest as one or more of the authors are employed by the funder of the study.

### III. Studies modelling of health and economic impact of vitamin D, folic acid, and iron interventions

An overview of the studies included and models used for modelling the impact of vitamin D [130–133], folic acid [122, 134–139] and iron [140–142] is shown in Table 3 and evaluation results in Table 4. Details for the individual studies are provided in the supplementary information.

### Intervention scenarios modelled, outcome focus, and applied modelling approaches

#### Vitamin D

In different studies, Markov microsimulation was used to model the preventive effect of fortifying bread [130] or dairy products [131–133] with vitamin D [131–133] or with vitamin D and calcium [130], on bone fractures in older women and related cost savings. Sandmann et al. [130] modelled the relative risk reduction in fracture incidence for different fracture types using an Excel spreadsheet comparative risk model. Two studies simulated transition through different fracture types based on age-related incidence and risk in older women [131] or older men and women [132, 133]. The health outcomes included were prevention of fracture incidence [130] or QALYs gained [131–133]. Economic outcomes were estimated considering costs related to residency-dependent health care, [130] and Incremental Cost Efficiency Ratio (ICER) [131–133].

#### Folic acid

Different scenarios of increasing folic acid intake of women of childbearing age were modelled; either via food fortification [134–139, 143–145], via folic acid supplements [134], or via campaigns to promote folate-rich foods or folic acid supplements [143].

In all studies, the avoided neural tube defect cases were modelled. Health outcomes included QALYs gained [134, 135, 138], or DALYs prevented [136, 137, 143]. Other health benefits modelled included myocardial infarct prevention [138] or health risks of excess folic acid intake in the general population [134, 136, 138]. Economic outcomes included the ICER [134, 143], cost-effectiveness [135, 137], and cost savings [138] related to the prevention of neural tube defects. In most studies, simple [144, 145] to more sophisticated [134–137, 139] decision-tree models were used to simulate the effect of folic acid food fortification on neural tube defects and related health and economic consequences, whereas in two publications the type of modelling was not made specific [138, 143].

#### Iron

In all three studies, the effect of iron-fortified foods on DALYs averted was modelled [140–142]. DALY’s were based on diarrhea-related morbidity, mortality as well as anemia-related IQ in young children [140–142]. Economic outcomes included fortification and societal costs (work productivity loss) per DALY averted [140, 141], loss of earnings [142], or fortification costs per DALY averted [142]. None of the studies clearly reported on the type of model and software used to model health and economic outcomes.

#### Quality of data input

##### Vitamin D

Quality of data input for these studies was rated as medium (Table 4). To model the health impact of improving nutrient intakes via fortified foods, preferably, well-established relationships between micronutrient intake, status, and health outcome are used. However, often these relationships are not well-established. For instance, the effect of vitamin D and calcium fortification on reduced risk for fractures was assumed to be the same as for supplements [130–133] whereas this reduced risk was only reported for supplements. Authors used best available evidence; for instance, hip and femoral fracture incidence and cost estimates were based on the national hospital databases, whereas non-hip fractures cases and medical cost estimates were based on literature [130–133].

##### Folic acid

Quality of data input for these studies was rated as medium or low (Table 4). Input data on folate intake and folic acid supplement intake, for instance, were selected from national survey data [134–136, 138, 139, 143] assuming that these were the same in other countries [143], or were not reported [137, 144, 145]. The neural tube defect prevalence or incidence rates were based on national data [134–139, 143–145]. One study described how the relation between increasing folic acid intake, serum folate concentration, and neural tube defect risk reduction was simulated [134]. Incidence rates of masking of vitamin B_12_ deficiency [134], myocardial infarct and colon/colorectal cancer [136, 138] were based on national data where available. Input data for costs related to a child with a neural tube defect, such as surgery, medical care and rehabilitation costs, were derived from diverse national and insurance databases [134, 135, 137, 138, 143].

##### Iron

The overall quality of data input for these studies was rated as low (Table 4). Studies used iron intake or prevalence of inadequate iron intake from surveys [142], prevalence of iron deficiency anemia from cohort studies [141], or assumptions were made on the prevalence of iron deficiency attributable to anemia due to lack of data on anemia [140]. For example, in one modelling study [141] reduced prevalence of anemia was converted into improved IQ based on data from a cohort study acknowledging lack of direct observations from intervention studies.

#### Robustness

##### Vitamin D

The model robustness of the studies on vitamin D, folic acid and iron was rated as medium or low (Table 4). The validation of the Markov microsimulation model [131–133] for vitamin D was reported elsewhere [146]. Validation of the Excel model however was not mentioned [130].

##### Folic acid

The model robustness of the folic acid studies was rated as medium or low (Table 4). In general, a decision tree model suffices to model one event as neural tube defects is a life-long disability without different stages of disease. For modelling of masking of vitamin B_12_ deficiency and colorectal cancer with different disease stages, a more complex model would have been more suitable [134, 136, 138].

##### Iron

The model robustness of these studies was rated as low (Table 4). In one modelling study [141], the simulated results were compared with observed data of the cohort study for internal validation, and with the data of previous studies for external validation, both showing good agreement. For the other two studies on iron, too little information was available to properly judge the robustness of the models.

#### Transparency

Transparency of the modelling was rated as variable for most vitamin D, folic acid, and iron studies (Table 4). Most publications were transparent in terms of what model and input data were used [134–137, 139, 144, 145], but few details were given of the model structure itself [130–132, 135, 138, 140–143].

#### Sensitivity analyses

Probabilistic, univariate, and deterministic sensitivity analyses were used. Deterministic one-way-sensitivity analysis was performed in most of the studies that simulated vitamin D [130–133] and folic acid fortification [135–138, 144, 145] (Table 4). In two iron studies, no information could be retrieved on sensitivity analysis performed [140, 147].

#### Data needs

Data needs depend on the type of models used. Considerable data and estimates were needed as inputs for the vitamin D model, e.g., inpatient and outpatient incident cases, risk reductions and costs per type of fracture. While generally, little data input was used for health-economic simulations of folic acid and iron interventions. For the latter, the models appeared to be self-built, and lack of overview of data input sources used made it hard to judge data needs (Table 4).

#### Accessibility

Although the TreeAge software described by five papers [131–134, 139] is accessible for use by others, the model structure is not. Also, the other used modelling tools were not accessible, which makes it impossible to reproduce these modelling studies.

#### Source of funding and conflict of interest

The authors of the health-economic modelling studies reported to have received either no funds [130] or public funding [134–139, 143–145] or industry funds [131, 132, 140] (Table 4). For one study the financial sources of the funding agency were not clear [133]. Disclosures reported financial support from SMB Belgium, [132] Danone [131], and Nestlé [140] who had no role in the design, conduct of the study, or interpretation of the data. Other authors had no conflict of interest to declare [134–139, 141–145].

## Discussion

The objective of this review was to obtain insights into the different modelling approaches that are currently used to estimate the health and/or economic impact of nutrition interventions aiming at reducing population intake of salt and sugars or increasing intake of micronutrients.The results showed that a wide variety of models have been used to estimate the health and economic impacts of changes in nutrient intakes, which makes it difficult to compare models and to draw clear conclusions about best practices.

The models included that evaluated the impact of micronutrient fortification or supplements were either simple decision tree or comparative risk models developed by the authors and looking at single health events without future change in the condition. The models varied widely in terms of applied approaches and methodologies.

The reviewed models evaluating salt or sugar and chronic diseases tended to be more complex as they involve intermediate risk factors and multiple disease states. Regarding the salt models, although they are very useful in determining the beneficial strategies for policy making, they had some limitations. e.g., in terms of using simplified assumptions to reduce complexity, difficulties in assessing the cost and benefits. Some were lacking country-specific data or had inequalities in terms of income in sampling.

The sugar models were even more complex as these were mainly models modelling the impact of taxes, which involves assumptions on impact of price elasticity on consumer behaviour. In addition, most of the models require specialized knowledge in order to be used and the more established models are often not directly publicly accessible. The quality of the studies seems to be very much dependent on both the model and the data used. For example, some studies using complex ‘big data econometric analysis’ to estimate price elasticities, used very simplistic analysis of calorie/weight changes [84, 118]. In contrast, studies using more standard health economic models, were based on very simple input parameters when it comes to price elasticities [102, 110].

Limited availability of data in lower income countries such as epidemiological data and cost data, may explain the relative low data input in the micronutrient modelling studies. Moreover, models simulating folic acid and iron interventions were less sophisticated than those for salt and sugar since 1) the relationship between intake and health outcomes is less well-established for iron and folic acid than for vitamin D, salt and sugar and 2) reducing salt and sugar reduction to reduce non-communicable diseases have received more public health attention than reducing micronutrient deficiencies.

A general finding was that the studies published in the last few years mostly performed better in terms of transparency, and sensitivity analyses. This might be explained by journals nowadays allowing authors to submit more detailed information as supplement to their manuscript. It should also be noted that differences in declaration of conflict of interest may be due to the journal where the study was published.

Several previous papers have also reviewed health modelling studies. In 2006, Unal et al. [148] performed a systematic review to evaluate the strengths and limitations of existing coronary heart disease health policy models. For example, authors concluded that these models have become more complex and comprehensive as a result of improving computer technology and wider usage, but also that the quality of the models has also improved over time so that more papers published the last few years tend to explicitly report assumptions, limitations, and sensitivity analyses. Also, Lhachimi et al. [24] evaluated different models for health impact assessment to explore whether already existing models can be used as a standard tool for health impact assessment. The conclusion was that none of the existing tools could serve as a standard, and that any tool that intended to fill this gap need to put equal weight on appropriate methodology and achievable data requirements also being end-user friendly. The study of Grieger et al. [149] included 45 simulation studies that modeled dietary strategies aiming to improve nutritional intake, body weight, and related chronic disease. In contrast to our review, where we classified studies on nutrients, they categorized the studies as modeling moderation, substitution, reformulation, or promotion dietary strategies. In line with our results, authors concluded that study quality was moderate to high. The study of Hendriksen et al. [40] was the first study that systematically compared various indirect and complex health modelling approaches for salt reduction based on four sets of key model features and their underlying assumptions and input data [40]. The authors concluded differences in model structure and input data may affect the health impact estimate, and that clearly defined assumptions and transparent reporting are crucial to be able to interpret the outcomes of a health impact assessment.

In line with our current review, these reviews made clear that the wide variety in modelling approaches makes it difficult to compare model outcomes, as the structural and methodological differences could have a major impact on the modeling results. In addition to these reviews, [148, 149] also the participants of our stakeholder workshop agreed that the outcomes of simulation modelling are highly dependent on the quality of the input data and assumptions used as model parameters. Therefore, to improve the quality judgement and comparison of increasingly complex modelling studies, the application of international health economic evaluation reporting standards, such as CHEERS, [150] is urgently needed.

Recently, Fattore et al. pointed out specific aspects that need to be emphasized when applying the international CHEERS standards in the reporting of economic evaluation of nutrition interventions [151]. For example, Fattore et al. recommend that the reporting of population characteristics should include e.g., socio-economic and behavioral factors, since these can have significant impact on the take-up and effectiveness of nutrition interventions [151]. In addition, specific emphasis should also be placed on the distribution of costs and savings between health, other sectors, and private individuals themselves to understand the relative cost burdens when considering the implementation of nutrition interventions. The reports of modelling studies should also include considerations of whether the setting in which the study was conducted influences the expected costs and health outcomes limiting the generalizability of the findings. In addition, different target audiences may require different health economic outcomes depending on the applied perspective to optimally inform multiple decision makers with different objectives. This is especially the case when cost and effect impacts are expected to fall on multiple sectors in the society [152]. Thus, further developments are needed on the international guidelines, such as CHEERS, to be applicable to nutrition modelling.

## Conclusions

This review shows that there is a wide variety of models used to estimate the health and economic impacts of changes in nutrient intakes, making it difficult to compare models and to draw clear conclusions about best practices. Models can yield useful insights for decision-making and have already been extensively used in policy making and resource allocation. Improved technology potentially increasingly enables practitioners and policy makers to use these models without necessarily understanding the inherent assumptions or data limitations [148]. However, health impact modelling in the domain of public health nutrition is still in its infancy, and there is substantial need for more nutrition-specific guidance development. This development could be supported by setting up an international research network aiming to develop modelling standards, open-source standard models, and compare nutrition health economic models both in terms of their structure and performance.

## Supplementary information

Supplemental information 1

Supplemental information 2

## Data Availability

All data generated or analysed during this study are included in this published article and its supplementary information files. Correspondence and requests for materials should be addressed to N.V. Venlet at publications@ilsieurope.be.
