# Supplementary material for: Modelling health and economic impact of nutrition interventions: a systematic review
Source: Eur J Clin Nutr. 2022 Oct 4;77(4):413–26. doi: 10.1038/s41430-022-01199-y (PMC10115624; doi:10.1038/s41430-022-01199-y)
Supplement: Supplementary file 1 — Supplemental information 1 [file 41430_2022_1199_MOESM1_ESM.docx]

**Search terms used and filters applied**

Search strings were developed for salt (RNU), sugar (MDK), vitamin D (MB).

**Salt**

***String used for PubMed:*** (salt [Title/Abstract] OR sodium [Title/Abstract] OR sodium chloride [Title/Abstract]) AND (reduction [Title/Abstract] OR reformulation [Title/Abstract] OR intake [Title/Abstract] OR consumption [Title/Abstract] OR tax [Title/Abstract]) AND (blood pressure [Title/Abstract] OR cardiovascular disease [Title/Abstract] OR stroke [Title/Abstract] OR health [Title/Abstract] OR disease risk [Title/Abstract] OR disability [Title/Abstract] OR quality of life [Title/Abstract] OR adjusted life years [Title/Abstract] OR health burden [Title/Abstract] OR public health [Title/Abstract] OR health impact [Title/Abstract] OR economic evaluation [Title/Abstract] OR economics [Title /Abstract] OR economic costs [Title/Abstract] OR health care costs [Title/Abstract] OR health care expenditure [Title/Abstract] OR cost effectiveness [Title/Abstract] OR cost analysis [Title/Abstract] OR cost benefit [Title/Abstract] OR cost utility [Title/Abstract] OR economic aspects of illness [Title/Abstract]) AND (simulation [Title/Abstract] OR estimation [Title/Abstract] OR modelling [Title/Abstract] OR scenario [Title/Abstract]) NOT (cohort [title] OR randomized [title] OR meta-analysis [title] OR meta-analysis [title] OR review [title] )

***String used for Scopus:***  (TITLE-ABS-KEY (salt  reduction  model)  AND  (public  health)  AND  (economic))  AND  PUBYEAR  > 2006  AND  (LIMIT-TO (LANGUAGE,  "English") AND  (LIMIT-TO ( DOCTYPE ,  "ar" ) )

**Sugar**

**String used for PubMed:** (((((sugar*[Title/Abstract] OR sucrose[Title/Abstract])) AND (reduction[Title/Abstract] OR reformulation[Title/Abstract] OR intake[Title/Abstract] OR consumption[Title/Abstract] OR tax*[Title/Abstract])) AND (obesity[Title/Abstract] OR overweight[Title/Abstract] OR dental[Title/Abstract] OR caries[Title/Abstract] OR health[Title/Abstract] OR disease risk[Title/Abstract] OR disability[Title/Abstract] OR quality[Title/Abstract] OR adjusted life years[Title/Abstract] OR health burden[Title/Abstract] OR public health[Title/Abstract] OR health impact[Title/Abstract] OR economic evaluation[Title/Abstract] OR economics[Title/Abstract] OR economic costs[Title/Abstract] OR health care costs[Title/Abstract] OR health care expenditure[Title/Abstract] OR cost effectiveness[Title/Abstract] OR cost analysis[Title/Abstract] OR cost benefit[Title/Abstract] OR cost utility[Title/Abstract] OR economic aspects of illness[Title/Abstract] OR cost saving[Title/Abstract])) AND (simulation[Title/Abstract] OR estimation[Title/Abstract] OR modelling[Title/Abstract] OR scenario[Title/Abstract])) NOT (cohort[Title] OR randomized[Title] OR meta-analysis[Title])

**String used for Scopus:** ((TITLE-ABS-KEY(sugar* OR sucrose) AND TITLE-ABS-KEY(tax*) AND TITLE-ABS-KEY(epi*) AND TITLE-ABS-KEY(health*)) AND PUBYEAR > 2006) OR (TITLE-ABS-KEY ( sugar  reduction  model ) )  AND  ( ( public  health ) )  AND  ( economic ) )

**Vitamin D**

**String used for PubMed:**

“Vitamin D” [Title/Abstract] AND (fortif*[Title/Abstract] OR enrich*[Title/Abstract]) AND (disease risk [Title/Abstract] OR disability [Title/Abstract] OR quality of life [Title/Abstract] OR adjusted life years [Title/Abstract] OR health burden [Title/Abstract] OR public health [Title/Abstract] OR health impact [Title/Abstract] OR economic evaluation [Title/Abstract] OR economics [Title /Abstract] OR economic costs [Title/Abstract] OR health care costs [Title/Abstract] OR health care expenditure [Title/Abstract] OR cost effectiveness [Title/Abstract] OR cost analysis [Title/Abstract] OR cost benefit [Title/Abstract] OR cost utility [Title/Abstract] OR economic aspects of illness [Title/Abstract]) AND (simulat* [Title/Abstract] OR estimat* [Title/Abstract] OR model* [Title/Abstract] OR scenario [Title/Abstract]) NOT (cohort [title] OR randomized [title] OR meta-analysis [title] OR review [title])

**String used for Scopus:** TITLE-ABS ( "Vitamin D" ) AND TITLE-ABS ( fortif* OR enrich* ) AND TITLE-ABS ( "disease risk" OR disability OR "quality of life" OR "adjusted life years" OR "health burden" OR "public health" OR "health impact" OR "economic evaluation" OR economics OR "economic costs" OR "health care costs" OR "health care expenditure" OR "cost effectiveness" OR "cost analysis" OR "cost benefit" OR "cost utility" OR "economic aspects of illness" ) AND TITLE-ABS ( simulat* OR estimat* OR model* OR scenario ) AND NOT TITLE ( cohort OR randomized OR meta-analysis OR review )

**Folic acid and Iron**

For folic acid, similar search strings for PubMed and Scopus as for vitamin D were used.

## **Eligibility criteria**

Studies that modelled the impact of modifying nutrient intake on public health and/or economic outcomes due to intervention (e.g., estimating potential changes in risk factor, disease incidence, disease burden, quality-/ disability-adjusted life years, or health care costs) were selected as eligible modelling studies. The search was limited to articles published in English from 2006 until August 2020. The filters applied in the search strategy excluded observational studies, randomized trials, meta-analyses, and review articles. In addition, publications were excluded that focused on:

- estimating disease burden due to current nutrient intakes
- estimating the number of subjects needed to treat to avoid nutrient deficiency
- modelling impact of nutrient supplementation
- modelling impact on nutrient intake (e.g. adequate intakes) solely without health outcome
- modelling impact of combined nutrient scenarios or nutrient indices, where it was not possible to disentangle the impact of one single nutrient
- modelling the impact on the environment (e.g. climate change, carbon footprint)
