# Supplementary material for: Modelling health and economic impact of nutrition interventions: a systematic review
Source: Eur J Clin Nutr. 2022 Oct 4;77(4):413–26. doi: 10.1038/s41430-022-01199-y (PMC10115624; doi:10.1038/s41430-022-01199-y)
Supplement: Supplementary file 2 — Supplemental information 2 [file 41430_2022_1199_MOESM2_ESM.pdf]

No	Nutrient	Author, journal	Year	Model type	Scenario's modelled	Outcome focus	(Credible) data input	Robustness	Transparency	Sensitivity/uncertainty analyses	Data needs	Accessability	Source of funding	Potential conflict of interest
1	salt	Aminde, et al. Open Hear. 2019	2019	Comparative risk assessment (Multicohort proportional multistate life table model)	Reduction in salt	Decrease in deaths attributable to cardiovascular diseases and diet-related cancers	High	High	Medium	Probabilistic	Considerable	Reproducible	Public	No
2	salt	Barton P et al. BMJ, 2011	2011	Comparative risk assessment	To reduce dietary salt (from 8.5 g/day by 3 g/day). Small decrease in population levels of BP or total cholesterol	Cardiovascular events avoided, QALY gained, savings in health care cost.	Medium	Medium	Low	Deterministic	Considerable	NA	Public	No
3	salt	Basu, et al. PLOS One, 2012	2012	Markov deterministic (mathematical modelling)	Salt reduction (target of 3 g/day -via a linear reduction in intake of 0.1 g/year- over 30 years)	Reduction in CVD (lowering blood pressure)	High	High	High	Different (multivariate)	Considerable	Reproducible	Not clear	Not clear
4	salt	Blakely, et al. Lancet Public Heal, 2020.	2020	Comparative risk assessment (Proportional Multistate lifetable)	Salt tax	Health gains (HALYs), health expenditure savings	High	High	High	Probabilistic	Considerable	Reproducible	Public	No
5	salt	Briggs, et al. BMC Health Serv Res, 2019	2019	Comparative risk assessment (PRIMEtime CE model validation, AdViSHE tool)	Reformulation	QALY	High	High	High	Probabilistic	Considerable	Reproducible	Public	No
6	salt	Bruins MJ, et al. Nutrients. 2015	2015	Comparative risk assessment (WHO calculation template)	Salt reduction (25% sodium reduction in soups	Number of years of life lost, years lived with disability, DALYs	High	High	High	Different	Little	Reproducible	Private	Yes
7	salt	Caro, et al. Food Policy, 2017	2017	Comparative risk assessment (Tax model, QUAIDS,modified version of the –nlsur- model)	Food tax	Impact on NCDs	Medium	Medium	Medium	Probabilistic	Modest	Reproducible	Public+Private	Yes
8	salt	Cobiac&Scarborough et al. BMJ, 2017	2017	Markov deterministic model (dynamic population)	Ideal risk reduction for salt consumption (WHO target)	Mortality and morbidity (years lived with disability) from NCDs that are averted or delayed.	High	Medium	Medium	Deterministic	Little	NA	Public	No
9	salt	Cobiac&Tam, et al. PLOS Medicine, 2017	2017	Comparative risk assessment (proportional multistate lifetable model)	Tax and subsidy, cost-savings to the health sector.	Health impact, cost-effectiveness, DALY	High	Medium	High	Different	Little	Reproducible	Not clear	Not clear
10	salt	Cobiac&Vos et. al. Heart, 2010	2010	Comparative risk assessment (proportional multistate life-table modelling)	Population health benefits and cost-effectiveness of interventions for reducing salt in the diet.	Health benefits and cost-effectiveness of salt reduction in the diet (Prevention of CVD, Cost effectiveness, DALY)	High	Medium	High	Probabilistic	Little	Reproducible	Public	No
11	salt	Collins&Mason et al. Value in Health, 2014	2014	Discrete event modelling (DES) (IMPACT CHD model)	Cost-effectiveness of 4 population health policies to reduce dietary salt intake to prevent coronary heart	Gain life-years and reduce health care expenditure.	High	High	High	Different	Modest	Reproducible	Public	No
12	salt	Coxson, et al. Hypertension 2013	2013	Discrete event simulation (DES), Shared modelling:	i) Gradual uniform 40% reduction in sodium consumption ; ii) Instantaneous 40% reduction in sodium consumption to achieve 2200 mg/d; iii) Instantaneous reduction to 1500 mg/d over 10 years.	Health benefits (CHD, CVD, stroke, hypertension induced deaths averted)	High	Medium	Medium	Different	Modest	NA	Public+Private	Not clear
13	salt	Dall TM, et al. Am J Health Promot, 2009a	2009	Decision tree	Potential long-term national productivity benefits from reduced daily intake of sodium.	Medical cost-savings with productivity increases.	High	High	High	Different	Modest	NA	Private	Yes

14	salt	Dodhia et al, J Hypertens 2012	2012	Comparative risk assessment	Impact of scaled-up reduction and impact of reduction in blood pressure.	Evidence-based interventions for prevention and treatment of blood pressure of blood pressure lead to health gains and cost-effectiveness.	High	Medium	High	Probabilistic	Considerable	Reproducible	Not clear	No
15	salt	Erkoyun E et al. Public Health, 2016	2016	Markov chain (DYNAMO health impact assessment tool)	Reduction of dietary salt intake (based to; reference, gradual decline, WHO advice)	Lower cardiovascular disease (burden) events	High	High	High	Different	Little	Reproducible	Public	No
16	salt	Fitzgeld, et al. BMJ Open, 2	2018	Comparative risk assessment (Probabilistic-Monte Carlo simulation)	Intervention (nutrition education and system-level dietary modification)	Improvement in health-related quality of life, reduction in absenteeism, measured in monetary amount	Low	Medium	Low	Probabilistic	Little	NA	Public	No
17	salt	Gillespie DOS et al., PLoS One 2015	2015	Discrete event simulation (DES) (IMPACT$_{SEC,}$validated CHD policy model)	Mandatory reformulation(reductions in salt content of 30% and 10%)	Reduce hypertension, inequality of CHD mortality.	Low	High	High	Probabilistic	Considerable	Reproducible	Not clear	No
18	salt	Goiana-Da Silva, World Health Org.Bull,2019	2019	Comparative risk assessment (PRIME)	Salt reduction	NCD mortality	High	High	Medium	Probabilistic	Considerable	Reproducible	Public	No
19	salt	Hendriksen MA, et al. PLoS One. 2015	2015	Markov chain (Dynamic Markov-type model-DYNAMO-HIA)	30% salt intake reduction (in different salt intake categories)	Health impact of salt reduction. Reduction of prevalence of IHD, stroke, and mortality	High	High	High	Different	Considerable	Reproducible	Public	No
20	salt	Hendriksen&Geleijnse, et al.PLOS One, 2017	2017	Markov deterministic (multistate transition model)	Salt reduction	Impact of salt reduction on morbidity and mortality. Mutual differences between the models.	High	High	High	Probabilistic	Considerable	Reproducible	Public	Not clear
21	salt	Hendriksen&Over, et al. J Public Health, 2018	2018	Comparative risk assessment (Health Impact modelling, DYNAMO-HIA)	Intervention	Health gains, gain in life expectancy	High	High	High	Different	Considerable	Reproducible	Public	No
22	salt	Hughes, et al., PLoS One 2015	2015	Discrete event simulation (DES) (IMPACT model)	Risk factor scenarios (smoking, physiacl activity, salt intake, saturated fat). Reduction in salt intake of up to 30%)	Reduction in CHD induced mortality	High	High	High	Different	Modest	Reproducible	Public	No
23	salt	Islek et al.BMC Public Health. 2016	2016	Markov model *(deterministic) (with the estimation of population level effects)*	Policy scenarios (baseline, ischemic stroke treatment, populaiton level policy). 30% salt intake reduction.	Reduction in ischemic stroke (IHD) and IHD mortality	High	High	High	Different	Considerable	Reproducible	Public	No
24	salt	Kontis V, et al. Lancet Glob Health. 2015	2015	Comparative risk assessment (Time-dependent population)	Imact of achieving targets for 6 risk factors. 30% reduction in salt intake.	Number of deaths delayed or prevented	High	High	High	Probabilistic	Considerable	Reproducible	Public	No
25	salt	Kontis V, et al. Lancet. 2014	2014	Comparative risk assessment (Time-dependent population)	Risk factor scenario. 30% reduction in salt intake.	Decrease in NCD premature mortality.	High	High	High	Probabilistic	Considerable	Reproducible	Public	No
26	salt	Kypridemos C et al.BMJ, 2017	2017	Discrete event simulation (DES) (IMPACT$_{NCD}$ microsimulation)	Counterfactual scenario (downward trend in salt consumption observed between 2001 and 2011	Impact and equity of salt reduction policies on primary prevention of cardiovascular disease (CVD) and gastric	High	High	High	Different	Little	Reproducible	Public	No
27	salt	Labonte, et al. PLOS One, 2019	2019	Comparative risk assessment (PRIME, health impact)	Traffic light labelleing	Improve NCD outcomes	High	High	High	Different	Considerable	Reproducible	Public	No
28	salt	Marklund, et al. BMJ, 2020.	2020	Comparative risk assessment	Replacement of discretionary salt (used at table or during cooking) with potassium	Prevent deaths	High	High	High	Probabilistic	Modest	Reproducible	Public+Private	Yes
29	salt	Martikainen, et al. EJCN, 2011	2011	Markov chain model (dynamic population structure)	To predict health economic consequences of modest reduction in salt intake (-1.0	Reduction in CVD burden (CVD events and mortality, quality-adjusted life years)	Medium	Medium	High	Deterministic	Modest	NA	Public+Private	Yes

30	salt	Mason H et al. PLoS One 2014	2014	Discrete event simulation (DES) (IMPACT CHD model)	Reduction of dietary salt intake (health promotion campaign, labelling, mandatory reformulation of	Reduction in CHD and gain in life-years (LYG),c ost saving	High	Medium	High	Different	Modest	Reproducible	Public	No
31	salt	Moreira PV, et al. PLoS One. 2015	2015	Discrete event simulation (DES) (IMPACT Food Policy model)	Reducing the consumption of processed and ultra processed foods (complete or partial replacement)	Reducing CHD disease burden.	High	High	High	Different	Considerable	Reproducible	Pubic	No
32	salt	Moreira, et al. Public Health Nutr. 2018	2018	Comparative risk assessment (impact food polciy)	Reduction in salt and other nutrients	Reduction in CVD burden (CVD events and mortality, quality-adjusted life years)	Medium	Medium	High	Probabilistic	Modest	Reproducible	Public	No
33	salt	Nghiem&Blakeley, et al. BMJ Public Health, 2016	2016	Markov model (deterministic) (macro-simulation model)	Intervention effects of salt reduction (salt substitution, limits on sodium in bread).	Health gains and cost savings, reduce ethnic inequalities in health.	High	High	High	Different	Little	Reproducible	Public	No
34	salt	Nghiem&Blakeley, et al. PLoS One. 2015	2015	Markov deterministic (macrosimulation model)	Eight different dietary salt reduction interventions	QALY, Cost-utility analysis. Health gains, reduce ethnic inequalities in health.	High	High	High	Different	Considerable	Reproducible	Public	No
35	salt	Ni Mhurchu et al. PLoS One. 2015	2015	Discrete event simulation (DES) (PRIME Macrosimulation model)	Health related food tax and subsidy on deaths prevented or postponed.	Number of deaths delayed or prevented	High	High	High	Different	Considerable	Reproducible	Public	No
36	salt	Nilson, et al. PLOS One, 2020.	2020	Comparative risk assessment (PRIME, Macro simulation)	Effect of compliance with WHO salt recommendations	Decreased deaths and costs associated with CVDs	High	High	High	Different	Considerable	Reproducible	Public	No
37	salt	O'Flaherty, et al., Bull WHO, 2012	2012	Comparative risk assessment (Spreadsheet model)	Policy scenario. Salt reduction. Conservative (1 g/d) and aggressive policy (3 g/day).	Reduced CVD and stroke induced mortality.	High	High	High	Probabilistic	Considerable	Reproducible	Public	No
38	salt	O'Keefe, et al. BMJ Open 2013	2013	Discrete event simulation (DES) (IMPACT), Food Policy Model	Potential reduction in CVD induced mortality by decreasing salt consumption.	CHD and stroke induced mortality	High	High	High	Probabilistic	Considerable	NA	Public	No
39	salt	Palar, et al. Am J Health Promot, 2009.	2009	Decision tree	Sodium reduction	Quality-adjusted life year (QALY) and health care cost saved, reduced hypertension prevalence.	High	Medium	High	Probabilistic	Considerable	NA	Public	Not clear
40	salt	Pearson-Stuttard et al.PLOS Med, 2018	2018	Markov individual (IMPACT Food Policy Model)	Reformulation (Optimal-100%, modest-50% compliance with 10 year target, pessimistic (100% compliance with 2 year target)	Cost-effectiveness, QALY, CVD deaths	High	High	High	Probabilistic	Considerable	Reproducible	Public	Yes
41	salt	Qin X, et al. Eur J Cardiovasc Prev Rehabil, 2009	2009	Comparative risk assessment	Impact BP lowering strategies	Lowering blood pressure and CVD incidence	High	Medium	High	Different	Modest	NA	Public	No
42	salt	Rubinstein, et al. BMC Public Health, 2010	2010	Comparative risk assessment (Epidemiological model)	Most of the interventions are cost-saving or cost-effective.	Potential years of life lost (PYLL), and DALY, cost saving, cost effectiveness	High	Medium	High	Probabilistic	Modest	NA	Public	No
43	salt	Saha, et al. Nutrients, 2019	2019	Comparative risk assessment (Macrosimulation, Monte Carlo Analysis)	Dietary intake (baseline, counte	Deaths attributable to cardiovascular diseases and diet-related cancers	High	High	High	Probabilistic	Considerable	Reproducible	Public	No
44	salt	Scarborough&Allender et al., EJCN 2012	2012	Markov chain (DIETRON model)	Baseline diets and counterfactual scenarios (decrease in salt intake).	CVD and cancer induced mortality.	Medium	Medium	High	Probabilistic	Little	NA	Public	No
45	salt	Scarborough&Nnoaham, et al. J Epidemiol Community Health, 2012	2012	Marcov chain (DIETRON model)	Reducing salt intake (1 g/day reduces the risk of CHD death by 12%).	Prevention of CVD and reduction of deaths.	High	Medium	Medium	Probabilistic	Little	NA	Public	No

	Nutrient	Author, journal	Year	Model type	Scenario's modelled	Outcome focus	(Credible) data input	Robustness	Transparency	Sensitivity/uncertainty analyses	Data needs	Accessibility	Source of funding	Potential conflict of interest
1	Sugar	Amies-Cull, BMJ 2019	2019	Markov model (multistate lifetable model)	comprehensive national program	Calorie, weight and BMI change, NCD incidence, QALYs, and healthcare costs	Medium	High	Medium	Probabilistic	Medium	Reproducible	Public	No
2	Sugar	Barrientos-Gutierrez, Plos One 2017	2017	Markov cohort model	SSB Tax	changes in BMI, prevalence of overweight, obesity and diabetes	Medium	Medium	Medium	Deterministic	Medium	Not reproducible	Public NGOs, University	No
3	Sugar	Basto-Abreu, Health Affairs 2019	2019	Markov cohort model	SSB Tax	obesity-related diseases, DALYs, QALYs, healthcare costs and projected savings	High	Medium	Medium	Probabilistic	Medium	Not reproducible	NGOs, Public and University	No
4	Sugar	Basu, Am J Public Health 2014 (a)	2014	Agent-based approaches	SBB tax	overweight, obesity, diabetes & health care cost saving	High	Medium	High	Probabilistic	Considerable	Reproducible	Not clear	No
5	Sugar	Basu, PLosOne 2014 (b)	2014	Discrete event modelling	SSB tax & cap-and-trade policy	overweight, obesity, diabetes	Medium	Medium	High	Deterministic	Considerable	Reproducible	Public	No
6	Sugar	Basu, Health affairs 2020	2020	Markov individual models (microsimulation) event history models	A Workplace Ban On Sugar-Sweetened Beverage Sale	QALYs saved and dollars saved from various diseases related to SSB consumption from employer and health care perspectives	Medium	Medium	Medium	probabilistic	Medium	Not reproducible	philantropiy and public. Some corporate links (KMPG)	Not specified
7	Sugar	Blakely, Lancet Public Health 2020	2020	Markov model (proportional multistate lifetable)	Subsidies, SSB Tax	HALYs and Health expenditure savings	High	High	High	Probabilistic	Medium	Reproducible	Public	No
8	Sugar	Bourke, BMJ Glob Health 2018	2018	multi-state life-table based Markov model	SSB Tax	number of overweight and obese, and diabetes prevention	Medium	Medium	High	Probabilistic	Medium	Reproducible	Public University of Queensland	No
9	Sugar	Briggs, BMC Public Health 2013	2013	Markov chain model	SBB tax	overweight, obesity	High	High	High	Probabilistic	Considerable	Reproducible	Public	No
10	Sugar	Briggs, BMJ 2013	2013	Markov chain model	SBB tax	overweight, obesity & tax revenu	High	High	High	Probabilistic	Considerable	Reproducible	Public	Not clear
11	Sugar	Briggs, Lancet Public Health 2017	2017	comparative risk assessment model	reformulation, price increase and change in market share	price changes and changes to SSB market share on obesity, dental caries, and type 2 diabetes	High	Medium	High	Probabilistic	Medium	Not reproducible	None	No
12	Sugar	Cleghorn, Preventive Medicine 2019	2019	Markov Model	cap on the size SSB	health benefits and cost-savings	Medium	Medium	Low	Deterministic	Medium	Not reproducible	Public	No
13	Sugar	Cobiac, Plos Med 2017	2017	comparative risk assessment model	Sugar & SBB tax	IHD, stroke, diabetes, hypertensive heart disease, osteoarthritis, several cancers & cost effectivness	High	Medium	High	Probabilistic	Little	Reproducible	Public	No
14	Sugar	Cobiac, Plos One 2016	2016	Markov chain model	Sugar intake reduction	CHD, stroke, diabetes, cancers of breast, colorectum, lung and stomach, kidney, liver and cirhosis	High	High	High	Probabilistic	Considerable	Reproducible	Public	No
15	Sugar	Crino, Nutrients	2017	Markov Model (multi-state, multiple cohort life table model)	cap on the size and reformulation	HALYs and economic gains	Medium	Medium	High	Probabilistic	Medium	Not reproducible	No specified	No
16	Sugar	Dharmasena, Health Economics 2012	2012	comparative risk assessment model	SBB tax	weight loss	High	Medium	High	Deterministic	Modest	Reproducible	Public	No
17	Sugar	Finkelstein Archives of Internal Medecine 2010	2010	comparative risk assessment model	SBB tax	weight and tax revenu	High	Medium	High	Deterministic	Modest	Reproducible	Public	No
18	Sugar	Finkelstein, Journal of Health Economics 2013	2013	comparative risk assessment model	SBB tax	weight	High	Medium	High	Deterministic	Modest	Reproducible	Public	no
19	Sugar	Fletcher , health Affairs 2010	2010	comparative risk assessment model	SBB tax and banning selling machines	weight	Medium	Low	Medium	Deterministic	Little	Not accessible	Public	No
20	Sugar	Ginsberg, Israel Journal of Health Policy Research 2017	2017	comparative risk assessment model	comprenhensive national program (sugar)	economic and health costs due to obesity and overweight, oral dental costs	Low	Low	Medium	NA	low	not reproducible	Not applicable	No

21	Sugar	Goiana da Silva, Plos Medicine 2020	2020	comparative risk assessment model	SSB tax	obesity incidence across different age groups	Medium	Low	Medium	NA	low	Not Reproducible (data is private)	Public	No
22	Sugar	Gortmaker, Am J Prev Med 2015	2015	Markov individual model (microsimulation)	SBB tax	BMI, DALYs, QUALYs & health care costs saving, tax revenu	High	High	High	Probabilistic	Considerable	Reproducible	Public	No
23	Sugar	Grummon, American Journal of Preventive Medicine 2019	2019	Markov individual model (microsimulation)	Warning labels	SSB intake, total energy intake, BMI, and obesity	medium	Medium	Medium	Probabilistic	Medium	not reproducible	None	Not specified
24	Sugar	Gustaven, Food Policy 2013	2013	comparative risk assessment model	Adjusting VAT	weight	Medium	Medium	Medium	Deterministic	Modest	Not accessible	Public	No
25	Sugar	Haby, Int J Obesity 2006	2006	Markov Model (deterministic)	Schoolbased programme to reduce SBB intake	DALYs and net incremental cost ratio	Low	Low	Low	Probabilistic	Modest	Not accessible	Not clear	Not clear
26	Sugar	Härkänen, Food Policy 2014	2014	comparative risk assessment model	SBB tax	weight, diabetes, CVD	Medium	Medium	High	Probabilistic	Modest	Reproducible	Public	No
27	Sugar	Hendriksen, Eur J Nutr 2011	2011	Decision tree model	Substiting sugar in soft drinks by sweeteners	weight, BMI	High	Medium	High	NA	Little	Reproducible	Not clear	No
28	Sugar	Huang, Circulation 2020	2020	Discrete event modelling	Sugar labeling and sugar labing + reformulation	cardiovascular disease and type 2 diabetes, QALYs, policy costs, health care, informal care, and lost productivity (health-related) savings and cost-effectiveness	High	Medium	High	Probabilistic	High	Reproducible	Public	No
29	Sugar	Huse, Epidemiology and Population Health 2019	2019	multiple-cohort Markov model	Mandatory restrictions on price promotions	cost-effectiveness, obesity-related health and cost impacts	Medium	HIgh	High	Probabilistic	Medium	Reproducible	Public	None
30	Sugar	Jevdjevic, Public Health 2019	2019	Markov model	SSB tax	oral health and caries-related economic burden	medium	Medium	high	Deterministic and probabilistic	Medium	not reproducible	None	None
31	Sugar	Kao, Economics and Human Biology 2020	2020	Markov cohort model	SSB tax	health and financial impact	Medium	High	High	Probabilistic	High	Reproducible	Public	No
32	Sugar	Lal, Nutrients 2020	2020	Markov (ACE model)	Reduction SBB consumption	weight, HALYs, healthcare cost savings	Medium	High	High (ref to earlier paper)	Probabilistic	High	Reproducible	Public	No
33	Sugar	Lal, PLOS Medicine 2017	2017	Markov cohort model	SSB tax	cost-effectiveness, health gains, and financial impacts	Medium	High	High	Probabilistic	High	Reproducible	Public	No
34	Sugar	Lee, American Journal of Preventive Medicine 2018	2018	Agent-based approaches	Warning labels	reduction in obesity prevalence	High	High	High	Probabilistic	High	Reproducible	Public	No
35	Sugar	Lee, Circulation 2020	2020	Markov individual event history models	SSB Tax	Health gains and economic savings	High	High	High	Probabilistic	Medium	Reproducible	Public	authors with corporate links
36	Sugar	Lin, Economics and Human Biology 2011	2011	comparative risk assessment model	SBB tax	weight	High	Medium	High	None	Modest	Reproducible	public	no
37	Sugar	Long, American Journal of Preventive Medicine 2015	2015	Markov Model (deterministic)	SBB tax	BMI, DALYs, QUALYs & healthcare cost saving, tax revenue	Low	Medium	Medium	Probabilistic	Modest	Not accessible	Public	no
38	Sugar	Long, Journal of Nutrion Education and Behavior 2019	2019	Discrete event modelling	SSB tax; ban of SSB from Supplemental Nutrition Assistance Program	health care cost savings, net costs and QALYs	High	High	High	Probabilistic	Medium	Reproducible	Public	No
39	Sugar	Ma, The Lancet Diabetes and Endocrinology 2016	2016	Markov Model	Sugar reduction in SBB	overweight, obesity, diabetes	Low	Medium	Medium	Probabilistic	modest	Not accessible	Public	no
40	Sugar	Manyema, BMC Public Health 2016	2016	Markov Model (using life tables)	SBB tax	stroke mortality	High	Medium	High	Probabilistic	Considerable	Reproducible	Public	No
41	Sugar	Manyema, PLosOne 2014	2014	comparative risk assessment model	SBB tax	obesity	High	Medium	High	Probabilistic	Modest	Reproducible	Public	No
42	Sugar	Manyema, PLosOne 2015	2015	Markov Model (using life tables)	SBB tax	diabetes & diabetes health care costs	High	Medium	High	Probabilistic	Considerable	Reproducible	Public	No
43	Sugar	Meijer, PlosOne 2015	2015	comparative risk assessment model	Sugar intake reduction	22 clinical enpoints incl. CVD, cancer, diabetes & health care costs saved	Medium	Low	Low	Deterministic	Modest	Not accesible	Public	no
44	Sugar	Mekonnen, Plos One 2013	2013	Markov Model (deterministic)	SBB tax	diabetes, CHD & mortality	Medium	High	High	Deterministic	Considerable	Reproducible	Public	No
45	Sugar	Nomaguchi, Health Policy 2020	2020	multi-state lifetable Markov model	SSB tax	health outcomes, healthcare costs, productivity gains	Medium	Medium	High	Probabilistic	High	Not reproducible	Public	No
46	Sugar	O'Neill, Annals of Epidemiology 2019	2019	comparative risk assessment model	SSB tax	type II diabetes incidence	Medium	Medium	Medium	Probabilistic	Medium	Not reproducible	Not specified	No

47	Sugar	Pearson-Stuttard, Plos Medicine 2017	2017	Discrete event modelling	National and targeted dietary policies	cardiovascular disease burden and disparities	Medium	High	High	Probabilistic sensitivity analysis. 1 SD variation per parameter.	High	Reproducible	Public	Authors with corporate links but no COI in relation to this study
48	Sugar	Penalvo, BMC Medicine 2017	2017	comparative risk assessment model	Subsidies, SSB Tax	CMD and disparities	Medium	Medium	Medium	Probabilistic	Medium	Not reproducible	Public	Authors with corporate links
49	Sugar	Ruff, Annals of Epidemiology 2015	2015	comparative risk assessment model	SBB tax	BMI, %body fat, overweight, obesity	Medium	Medium	High	Deterministic	Modest	Reproducible	Public	no
50	Sugar	Salgado, PLOS Medicine 2020	2020	Markov cohort model	Reduction in SSB consumption	diabetes, cardiovascular diseases (CVDs), and mortality among adults	medium	medium	High	Probabilistic	High	Reproducible	Public	No
51	Sugar	Sanchez-Romero, PLosMed 2016	2016	Markov Model (deterministic)	SBB tax	diabetes, CVD incidence and mortality	Medium	High	High	Probabilistic	Considerable	Reproducible	Public	No
52	Sugar	Scheelbeek, BMJ 2019	2019	comparative risk assessment model	20% price increase in high sugar snacks	changes in weight, BMI, overweight, obese and prevalence of obesity.	medium	Medium	Medium	Probabilistic	Medium	Not reproducible	Public	No
53	Sugar	Schwendicke, BMC Public Health 2017	2017	comparative risk assessment model	SSB tax	overweight and obesity	Medium	Medium	Low	Probabilistic	Medium	Not reproducible	Public and authors	No
54	Sugar	Sowa, The European Journal of Public Health 2018	2018	Markov Model	SSB tax	oral health and costs of dental care	Medium	Medium	Low	Probabilistic	Medium	Not reproducible	Public University	No
55	Sugar	Summan, BMJ Global Health 2020	2020	mathematical models	SSB tax	years of life gained (YLG), (premature) deaths averted, change in consumer spending and change in tax revenue	Medium	Low	Low	Deterministic	Medium	Not reproducible	Public	Employee of the public funding organization
56	Sugar	Urwannachotima, BMC Oral Health 2020	2020	complex System dynamics modelling	SSB tax	dental caries	Low	Medium	Low	Deterministic	High	Not reproducible	Public Thai	None
57	Sugar	Vecino-Ortiz, Social Science & Medicine 2018	2018	comparative risk assessment model	SSB tax	overweight and obesity	Medium	Medium	Medium	Deterministic	Medium	Not reproducible	Public University	Not specified
58	Sugar	Veerman, Plos One 2016	2016	Markov Model (deterministic)	SBB tax	IHD, stroke, diabetes, hypertensive heart disease, osteoarthritis, several cancers & cost effectivness	High	High	High	Probabilistic	Considerable	Reproducible	Public	Yes
59	Sugar	Wang, Health affairs 2012	2012	Markov Model (deterministic)	SBB tax	BMI, diabetes, CVD & tax revenu	Low	Medium	Medium	Probabilistic	Modest	Not accessible	Public	no
60	Sugar	Wilde, American Journal of Public Health 2019	2019	Markov individual models (microsimulation) event history models	SSB tax (penny per ounce)	healthcare and societal perspective, cost savings, health gains, tax paid, out of pocket health care savinbgs, ICER, QALYs, net costs for industry under different pass through rate, tax revenues for government and health care savings	Medium	High	Medium	Probabilistic	High	Reproducible	Public	No but two author receivers personal fees from other public/private sector
61	Sugar	Zheng, Nutrients 2019	2019	comparative risk assessment model	SSB substitution	BMI, waist circumference (WC), and overweight status in early adulthood	Medium	Low	Low	Deterministic	Medium	Not reproducible	public University	No

	Nutrient	Author, Journal	Model type	Health outcome	Economic outcome	(Credible) data input	Robustness	Transparency	Sensitivity/uncertainty analyses	Data needs	Accessibility	Source of funding	Conflict of interest
1	Iron	Plessow PLoS One. 2016	Stepwise approach using Popultaion Attributable Fraction (PAF) = Simulated Decision Tree (SDT)	DALYs from literature	Price sensitivity<br>Net social costs per DALY averted	Low	Low	Low	Deteministic	Low	Not accessible	No	No
2	Iron	Fiedler Food Nutr Bull. 2013	Unclear	Changes in prevalence of inadequate intake<br>Changes in incidence of inadequate intake<br>DALYs averted	Cost per reduction in the prevalence of inadequate intake<br>Cost per DALY averted	Low	Low	Low	Deteministic	Low	Not accessible	Yes	Yes
3	Iron	Sharieff Int J Technol Assess Health Care. 2008	Monte Carlo: Simulated Markov Model	Mortality averted	Costs per DALY<br>Benefit:cost ratio	Low	Medium	Low	Probabilistic	Low	Not accessible	Yes	Yes
1	Folic acid	Rabovskaja J Nutr. 2013	Decision tree	quality-adjusted life-years (QALYs), lifeyears gained (LYG), avoided NTD cases, and additional severe neuropathy cases	Incremental Cost-Effectiveness Ratio (ICER)	Medium	Medium	High	Probabilistic	Low	Not accessible	High	High
2	Folic acid	Dalziel Public Health Nutrition 2010	Unclear	Incremental Cost-Effectiveness cost-utility analyses, expressed as cost per DALY averted	Incremental Cost-Effectiveness cost-utility analyses, expressed as cost per DALY averted	medium	Low	Low	Deteministic	Low	Not accessible	High	High
3	Folic acid	Bentley Public Health Nutrition 2008	authors used either published estimates or a Markov modelling	QALYs gained	Cost saving	medium	Low	Low	Deteministic	Low	Not accessible	High	High
4	Folic acid	Jentink Eur J Public Health. 2008	Decision tree	numbers of NTD cases prevented<br>Life Years gained<br>QALY gained	Cost-effecttiveness	medium	Low	Low	Deterministic	Low	Not accessible	High	High
5	Folic acid	Hoekstra Food Chem Toxicol. 2008	Unclear	Change in incidence<br>Change in DALY	NA	Medium	Low	Medium	Deterministic	Low	Not accessible	High	High
6	Folic acid	Llanos Health Policy. 2007	Self-built Decision tree	numbers of NTD cases prevented, fetal and infant deaths prevented, DALYs prevented	Cost-effectiveness-ratio (Costs/DALY averted)	High	Medium	High	Deterministic	Low	Accessible	High	High
7	Folic acid	Hoddinott Ann N Y Acad Sci 2018	Self-built Decision tree	NTD deaths prevented, DALYs prevented	Cost-effectiveness-ratio (Costs/DALY averted)	Low	Low	High	Deterministic	Low	Not accessible	High	High

8	Folic acid	Kancherla Birth Def Res 2018	Decision tree	numbers of NTD cases prevented, and deaths prevented	NA	Low	Low	Medium	None	Low	Not accessible	High	High
9	Folic acid	Saing Appl Health Econ Health Pol 2019	Decision tree	numbers of NTD cases prevented<br>Life Years gained<br>QALY gained	Incremental Cost-Effectiveness Ratio (ICER)	Medium	Medium	High	Probablistic	Low	Not accessible	High	High
1	Vitamin D	Hiligsman 2015	Markov individual model (microsimulation)	Prevented incidence of fractures<br>QALY gained<br>Life Years Gained	Incremental Cost-Effectiveness Ratio (ICER)	Modest	Medium	Low	Deterministic	High	Not accessible	Private funding	yes
2	Vitamin D	Sandmann Public Health Nutr. 2015	Spreadsheet model	Prevented incidence of fractures	Costs prevented	Modest	Medium	Low	Deterministic	High	Not accessible	Not clear	No
3	Vitamin D	Ethgen Osteoporos Int. 2016	Markov individual model (microsimulation)	QALYs gained	Incremental costs per QALY<br>Incremental Cost-Effectiveness Ratio (ICER)	Modest	Medium	Medium	Probabilistic	High	Not accessible	Public and private	Yes
4	Vitamin D	Hiligsmann Exp Rev Pharmaecon & Outc Res 2018	Markov individual model (microsimulation)	Prevented number of fractures, QALY gained<br>Life Years Gained	Incremental costs per QALY<br>Incremental Cost-Effectiveness Ratio (ICER)	Modest	Medium	Low	Deterministic	High	Not accessible	Not clear	No
